# Study of the Potential Accumulation of the Pesticide Alpha-Endosulfan by Microplastics in Water Systems

**DOI:** 10.3390/polym14173645

**Published:** 2022-09-02

**Authors:** Sílvia D. Martinho, Virgínia Cruz Fernandes, Sónia A. Figueiredo, Cristina Delerue-Matos

**Affiliations:** REQUIMTE/LAQV—Instituto Superior de Engenharia do Porto do Instituto Politécnico do Porto, 4249-015 Porto, Portugal

**Keywords:** microplastics, pesticides, adsorption, wastewater

## Abstract

Microplastics (MP) are spread into all ecosystems and represent a threat to the equilibrium of the environment and human health, not only due to their intrinsic characteristics but also to their action as effective carriers of contaminants, such as pesticides, pharmaceuticals, polychlorinated biphenyls and polycyclic aromatic hydrocarbons. The pesticide α-endosulfan is persistent and spread in the environment. The MP are another possible way of dissemination to be considered in the fate of this pesticide. The adsorption dynamics of α-endosulfan by six different MP (low-density polyethylene—LDPE, polyethylene-co-vinyl acetate, unplasticized polyvinyl chloride, polyamide 6, polystyrene granule, polypropylene granule) with different sizes/shapes and chemical compositions were evaluated. The most critical situation was identified for the system LDPE (particle size < 300 μm). Equilibrium studies (48 h equilibrium time) were performed for distilled, tap and filtered river water. Based on the Langmuir model parameters, the highest maximum adsorption capacity was obtained for distilled water, followed by filtered river and tap waters (i.e., 366 ± 39, 247 ± 38, 157 ± 22 μg/g). The obtained results demonstrate the important role that microplastics may have in the fate and transport of pesticides and their potentially harmful effect on the environment, which requires further investigation.

## 1. Introduction

Microplastics (MP) are defined as plastic particles with a size of less than 5 mm, which are widely spread into the terrestrial and aquatic environment [1]. MP can be provided by two sources: primary production in the industries, and secondary, resulting from the fragmentation of large pieces of plastics [2,3]. In recent years, researchers had been studying the consequences of the excessive use of plastics and their incorrect disposal and they were able to prove the widespread presence of MP in surface waters [4,5], fresh waters [5,6,7,8], bottled water [9], wastewaters [4,5,10,11,12], sea-ice in the Arctic and Antarctic [13], seabed sediments [13], sand beaches [4,14], soils [3,15], several species of living organisms (e.g., seabirds, crustaceans, fish, gobies, barnacles, turtles, seals, earthworms) [16,17,18], food products (e.g., salt and honey) [9,19,20], and also in indoor and outdoor air [4,21,22]. Moreover, recent reports demonstrate that MP can act as a vector for environmental contaminants. There are studies demonstrating that contaminants such as polycyclic aromatic hydrocarbons (PAHs), polychlorinated biphenyls (PCBs), dioxin-such as chemicals, polybrominated diphenyl ethers (PBDEs), toxic metals, pharmaceuticals, and pesticides [3,7,15,18] are adsorbed and transported by MP.

Although the consequences of the presence of MP in the environment are not fully understood, namely the acute and chronic effects in humans, some studies report the ingestion, accumulation, and toxicity effects of MP on living organisms and the possible effects that can be projected, such as decreasing the gut microbial community, affecting the reproduction and avoidance behaviors of springtails, changing the energy metabolism, lowering the locomotor behavior, decreasing the body length of nematodes, inhibiting the food system and excretion of snails and affecting the oxidative stress [7,16,18,23]. The ingestion and harmful effects of MP, including inflammation, malnutrition, and changes in reproductive behavior, neurotoxic effects, lipid oxidative damage and hepatic stress, have been reported in aquatic organisms, such as fish, prawns, shrimps, mussels, oysters, sea cucumber, and zooplankton [24,25,26,27,28,29,30,31,32,33,34,35].

A potential negative effect on human health can be predicted once that big part of the aquatic organisms is directly connected to the food chain [25], and it is reported that the persistent presence of MP leads to oxidative stress, cytotoxicity, translocation to other tissues, and chronic inflammation. Also, the risk of cancer and the increase of immune or neurodegenerative diseases has been connected to this persistent pollutant [4,7,16,36]. 

The exploration of the activities that have more influence on this emergent pollution problem must be the priority if the goal is to minimize the several impacts that have been seen in recent years. Agriculture has been described as having a relevant impact on the contamination of soils with MP. Plastic mulching and other plastic wastes and irrigation through plastic tubes are considered important sources of MP in agricultural activities [37,38]. Techniques such as the application of sewage sludge to fertilization of the soil have been reported as another relevant source of contamination, with an estimated 125–850 t of MP per million habitants added to the Europe soils each year by the farmlands [39]. Once there is the possibility of interaction between them MP and pesticides, their simultaneous dispersion can represent one of the biggest problems to the environment, resulting in contamination and dissemination of the pollutants in water, sediments, and organisms, with possible adverse effects on the ecosystems.

Pesticides are widely used in agriculture to control different ill-effects of pests and improve production yield. Although the advantages are appealing, their persistence in the environment can be a concern affecting human health and the aquatic and terrestrial ecosystems [40,41]. Endosulfan is an organochlorine (OC) pesticide that had been extensively used in the past with a huge effective actuation to a broad number of insects, pests and mites [42] and it appears to be one of the most stable pesticides known. However, endosulfan has been considered an endocrine disruptor and contributes adversely to human health in several paths, such as physiological disorders, inducing seizures or cancer development [43]. Unlike other pesticides, this in particular demonstrated its persistence during the time, for example, of the monitoring of air around the Laurentian Great Lakes between the 1990s and 2000s, which did not show any attenuation of the presence of endosulfan [44]. Consequently, the use of this organochlorine pesticide has been prohibited or restricted in several countries, and it had been included in the Stockholm Convention as a part of the list of Persistent Organic Pollutants (POPs) [45]. Also, its low solubility in water increases the tendency to be adsorbed in soils and sediments, and the potential to be accumulated in the fat tissues of living organisms. Once that endosulfan is persistent and dispersed in the environment [46,47,48], its interaction with MP can predict a real example of harmful pollutant dispersion in the environment. Therefore, this pesticide has been selected to be the focus of this study. 

Although several authors have studied the interactions of the pollutants with MP [49,50,51], due to the importance of understanding the infinite possible combinations, this topic should be further explored. The study of adsorption (kinetics and equilibrium) of pesticides onto the MP is a topic to be developed, in order to understand their fate and transportation [52]. The kinetic and equilibrium studies provide useful information about the processes that may occur between the MP-toxic chemical. Plastic types, size, color, and physical and chemical properties are characteristics that can interfere directly with the sorption behavior [1,3,53]. The models that are applied to these studies can help to predict the type of sorption involved: physical and/or chemical sorption. Physical sorption occurs when attraction forces are involved (e.g., van der Waals forces), and due to the weak bonds formed it can be a reversible process. When stronger chemical bonds are involved the process is considered irreversible [54]. 

To the best of our knowledge, adsorption studies with several MP—low-density polyethylene (LDPE), polyethylene-co-vinyl acetate (EVA), unplasticized polyvinylchloride- (UPVC), polyamide 6 (PA6), polystyrene (PS) and polypropylene (PP)—with different shapes and sizes, and the organochlorine pesticide α-endosulfan, in aqueous solutions, are not yet described in the literature. These MP had received growing concern as environmental pollutants because of their impact on the ecosystems [55]. Therefore, this work aims to study the interactions between the different microplastics (different shape/size and chemical composition) and the pesticide α-endosulfan in aqueous solution (distilled, tap and river waters) and understand the possible interaction and dispersion/fate that can occur in the environment. The influence of the aqueous matrix (distilled, tap and river waters) on the sorption behavior of the system MP-pesticide will be evaluated. This research also involved the optimization of the extraction conditions and validation of the analytical method with better extraction efficiency to ensure a correct evaluation of the concentration of the α-endosulfan in aqueous solution. Also, stability and homogenization tests of the pesticide solution were performed.

## 2. Materials and Methods

### 2.1. Reagents and Solutions

Analytical-standard α-endosulfan with high purity (≥98%) was obtained from Sigma Aldrich-Merck (Darmstadt, Germany). Chromatography-grade n-hexane was purchased from Merck (Darmstadt, Germany). Analytical-grade ethyl acetate from Dasit Group (Val de Reuil, France) and dichloromethane from Sigma Aldrich-Merck (Darmstadt, Germany) were used. Stock solutions of the pesticide were prepared in n-hexane (10,000 µg/L) and were kept in darkness and refrigerated at 4 °C. Working-standard solutions were prepared by appropriate dilution of the stock solutions in n-hexane.

MP were supplied by Goodfellow (Hamburg, Germany) and their characteristics, such as particle information and experimental concentration, are presented in Table 1. 

### 2.2. Samples

Distilled water (pH 5.95) was obtained from a Milli-Q water purification system from Millipore Vent filter MPK01 (Simplicity 185, Millipore, Molsheim, France). 

The tap water (pH 6.98) was collected in REQUIMTE/LAQV laboratory in Porto, Portugal (GPS 41.1815, −8.5937). The river sample (pH 7.22) from Douro River, Portugal. (GPS 41.1401, −8.6167) was collected in a glass bottle and was filtered (nylon filter (Membrane Solutions, Auburn, WA, EUA) with a pore size 0.45 μm) to remove suspended solids. 

### 2.3. Procedure

#### 2.3.1. Optimization of the Liquid-Liquid Extraction of α-Endosulfan from Aqueous Solution

Each aliquot of 1 mL of the standard solution of α-endosulfan (150 µg L^−1^) in distilled water was placed in a 20 mL glass flask (Linex - Vilabo, Marinha Grande, Portugal) and an aliquot of 1 mL of the extraction solvent was then added into the flask in the proportion of 1:1 (performed in duplicate). In order to optimize the best conditions for the extraction of α-endosulfan from aqueous solution, several extraction solvents (n-hexane, ethyl acetate, and dichloromethane), immiscible in water, were tested. The mixture was then vigorously shaken on a vortex agitator (VWR- Analog Vortex mixer, Radnor, PA, USA) for 10 min. The formation of fine droplets during the stirring process facilitated the contact of the α-endosulfan with the extraction solvent. The separation of the two phases occurred spontaneously in the glass flask after standing for a few minutes and the extraction solvents were left scattered on the upper layer of the solution in the case of n-hexane and ethyl acetate, and the lower layer in the case of dichloromethane. With a micropipette (VWR, Radnor, PA, USA), a volume of 500 µL of the organic phase was transferred to another glass flask and filtered with a PTFE filter of 0.22 µm (BGB Analytik, Böckten, Switzerland). 

After the filtration, the extract was injected into a gas chromatograph (GC) with an electron capture detector (ECD) to determine the concentration of α-endosulfan, as described in Section 2.3.2. The extractions were performed in duplicate and the analysis in triplicate. The extraction efficiency was evaluated in order to determine the best extraction solvent.

The evaluation of the extraction efficiency was performed by the calculation of the extraction efficiency (R%) according to Equation (1):

(1) $$R\%=C_{ext}/C_{0\;}\times 100,$$

where the C_ext_ (µg·L^−1^) and C_0_ (µ·L^−1^) are the extracted concentration and initial concentration of α-endosulfan, respectively.

#### 2.3.2. GC Analysis 

The gas chromatograph (GC-2010, Shimadzu, Kyoto, Japan) was used for the determination of α-endosulfan concentration. A volume of 1 µL of the sample was injected through a splitless mode, using an injector temperature of 250 °C. The starting temperature of the column oven was 40 °C and the temperature was kept for 1 min. The temperature was increased to 290 °C at a rate of 20 °C/min and then kept for 3 min. Between the column and the ECD, high purity nitrogen make-up gas from Nippon Gases (Maia, Portugal) was added at a rate of 30 mL·min^−1^. The temperature used in ECD was 300 °C. The operating system was executed by Shimadzu’s GC Solution software (Kyoto, Japan). A column Zebron-5MS from Phenomenex (Madrid, Spain) (with dimensions 30 m × 0.25 µm i.d. × 0.25 mm) was used. The calibration curve was performed with standard solutions of α-endosulfan with a range of concentration between 20 to 175 μg·L^−1^. To validate the method, standard solutions of 20 and 175 μg·L^−1^ were injected 10 times to evaluate the reproducibility of the results. 

#### 2.3.3. Stability and Homogenization Tests of α-Endosulfan Aqueous Solutions

To study the stability of the α-endosulfan aqueous solutions, the influence of three parameters was evaluated: time and temperature of storage of the solutions, and type of agitation used for homogenization. The samples were collected (in duplicate) and subjected to the same procedure of pre-treatment and analysis (in triplicate), as described in Section 2.3.1 and Section 2.3.2, respectively.

To study the effect of storage time, samples of the aqueous solutions of α-endosulfan (150 µg·L^−1^) were collected immediately after finishing the solution preparation, after 24 h of rest and after 48 h.

For the evaluation of temperature influence, the control samples of α-endosulfan were prepared with a concentration of 150 µg·L^−1^ and stored at variable (15–20 °C) and controlled (20 °C) temperatures. The solutions were kept in these conditions for 96 h and aliquots of 1 mL were collected every day. 

The effect of solution homogenization performed by orbital and vortex shaking was tested. Samples were collected in the beginning (after solution preparation) and after 4 h of constant orbital stirring, with the difference of using orbital shaking before collecting the samples for the second test. Aliquots of 1 mL were collected from the glass Erlenmeyer flasks (Linex - Vilabo, Marinha Grande, Portugal) subjected to constant orbital stirring at 110 rpm (Orbital shaker AO-400, Busen, Madrid, Spain). The vortex agitation (VWR- Analog Vortex mixer) of the glass Erlenmeyer flasks was performed for 1 min before collecting 1 mL of sample. 

#### 2.3.4. Preliminary Evaluation of Adsorption Affinity of Six Systems Microplastics/α-Endosulfan

A 100 mL volume of α-Endosulfan aqueous solutions (150 µg·L^−1^) was poured into glass Erlenmeyer flasks to weighted amounts of the six MP selected for this study, LDPE, EVA, UPVC, PA6, PS and PP (corresponding to a concentration of 1 g·L^−1^). The initial samples were taken before the contact with MP and the blank experiments (without MP) were performed in parallel. After a contact period of 48 h under orbital stirring at 110 rpm (Orbital shaker AO-400, Busen), the final samples (aliquots of 1 mL) were collected, then homogenized in a vortex agitator for 1 min and filtrated with a PTFE filter (0.22 µm pore size diameter) to a glass flask. The extraction with n-hexane was performed as described in Section 2.3.1. All the samples were collected in duplicate and injected in triplicate in the GC (Section 2.3.2). The evaluation of the removal efficiency was calculated by the same formula as for the recovery efficiency (Equation (1)) to compare the different behaviors of MP.

#### 2.3.5. Batch Adsorption Experiments

Kinetic batch adsorption experiments were performed to study the equilibrium time and adsorption rate of α-endosulfan into LDPE. One weighted portion of 50.0 mg of LDPE was placed in a glass Erlenmeyer flask of 100 mL. A 50.0 mL volume of aqueous solution of α-endosulfan (150 µg·L^−1^), prepared with distilled water, was put in contact with LDPE for 48 h with constant orbital stirring at 110 rpm (Orbital shaker AO-400, Busen). Initial samples were taken (in duplicate) to measure the initial concentration of α-endosulfan. Aliquots of 1 mL (in duplicate) were collected at defined time intervals, after 1 min of agitation on the vortex and filtration with a PTFE filter of 0.22 µm in a glass flask, then following the procedure described in the previous section for extraction and analysis (Section 2.3.4).

For the equilibrium experiments, 6 different quantities of LDPE were weighted, in duplicate, with concentrations between 0.30 to 1.40 g·L^−1^, into 50 mL glass Erlenmeyer flasks with 10 mL of α-endosulfan aqueous solution with the same concentration (150 µg·L^−1^) that was used in the kinetic experiments. The initial sample was collected in duplicate mL (aliquots of 1 mL). The Erlenmeyer flasks were subjected to constant orbital stirring at 110 rpm for 48 h. In the end, the glass Erlenmeyer flasks were agitated on a vortex and the final samples were collected in duplicate, followed by the extraction procedure and analysis previously described (Section 2.3.4). The equilibrium studies were performed for three types of aqueous matrix: distilled, tap and (filtrated) river water.

In parallel to the kinetic and equilibrium experiments, blank (without MP) experiments were performed, and samples were taken in duplicate at the beginning and the end of the experiments. All experiments were carried out at room temperature (20 °C). The same procedure of extraction and analysis was followed (Section 2.3.4).

The adsorption capacity of the microplastic under study was calculated according to Equation (2) for the kinetic studies:

(2) $$q_{t}=\left(C_{0}{-C}_{t}\right)\cdot V/m,$$

where C_0_ (µg·L^−1^) is the initial concentration of α-endosulfan and Ct (µg·L^−1^) is the concentration of solution at time (t), V (L) is the volume of α-endosulfan solution at that time and m (g) is the microplastic mass. For the equilibrium studies, the same equation was used considering the time (t) as the end of the experiment, corresponding to the equilibrium time.

## 3. Results and Discussion

### 3.1. Liquid-Liquid Extraction of α-Endosulfan with Different Solvents

The selection of an appropriate extraction solvent is important to ensure the best extraction efficiency of the pesticide under study (α-endosulfan) from the aqueous solution. The solvents studied were selected considering the solubility of the α-endosulfan in the respective solvent and its immiscibility in water. This pesticide presents a solubility of approximately 24 g·L^−1^ for n-hexane and higher than 200 g·mL^−1^ for ethyl acetate and dichloromethane at 20 °C [56]. It is known that α-endosulfan presents a lower solubility in water, approximately 0.33 mg·L^−1^, which is not influenced by the pH value between 5 to 9, the pH range of this study [56].

Table 2 presents the results of the extraction efficiency obtained using tree organic solvents.

According to the results, all the solvents presented an acceptable efficiency in extracting the pesticide from the water, however, there are differences in the recovery ratio of solvents. The n-hexane was chosen since it presented the highest extraction efficiency (95.1% with 1% RSD) for α-endosulfan, and also good chromatographic performance. Another advantage of using this solvent is the possibility of simplifying the experimental process by eliminating the evaporation step that would be necessary for the other solvents. The determination of the calibration curve, limits of detection (LOD) and quantification (LOQ) were performed for the GC-ECD analysis of α-endosulfan. The coefficient of determination (R^2^) was 0.9927, demonstrating the linearity of the calibration curve. The LOD and LOQ obtained for α-endosulfan were, respectively, 16.0 and 53.2 µg·L^−1^. All the samples were collected and extracted in duplicate, analyzed in triplicate and subjected to a validation of a maximum relative standard deviation of 5%.

### 3.2. Study of Stability of and Homogenization of α-Endosulfan Aqueous Solutions

To understand the behavior of α-endosulfan in aqueous solutions, stability studies were performed. 

To test the effect of storage time, samples were collected after their preparation, 24 h and 48 h after. In the samples with 24 h and 48 h of resting time, similar concentrations were measured, unlike the initial aliquots taken immediately after preparation, which presented a lower concentration. These experiments were performed in duplicate, and it was possible to conclude that a resting period of 24 h is recommended to ensure the complete dissolution of the pesticide. The stability of α-endosulfan solutions was verified for at least 48 h. 

The influence of temperature was tested using α-endosulfan solutions prepared with 24 h of rest. The effect of room (15–20 °C) and controlled (20 °C) temperatures was tested for 96 h. It was possible to observe that all the daily samples collected in duplicate kept a stable concentration in both temperature tests. No difference was observed between room temperature solutions and controlled temperature solutions. The stability at room temperature was demonstrated for 96 h.

It is important to test the homogenization of solutions (orbital shaking and vortex agitation) because the α-isomer of endosulfan has a low solubility capacity in water [44]. All solutions were kept in the orbital shaker at 110 rpm for 4 h, until the aliquot collection. The samples collected after orbital shaking presented a 12% variation of concentration between the replicates. The use of the vortex agitation after collecting the aliquots, to increase homogenization of the solutions in the glass Erlenmeyer flasks, has shown to be effective, resulting in a 1% variation between the replicates. 

Considering the results obtained, all the further experiments were performed using a 24 h resting period at room temperature after the preparation of the α-endosulfan solutions, and the vortex shaker was used to provide the homogenization of the solutions before collecting the samples. 

### 3.3. Preliminary Studies of Adsorption Affinity of Six Microplastics/α-Endosulfan Systems

**Figure 1:**
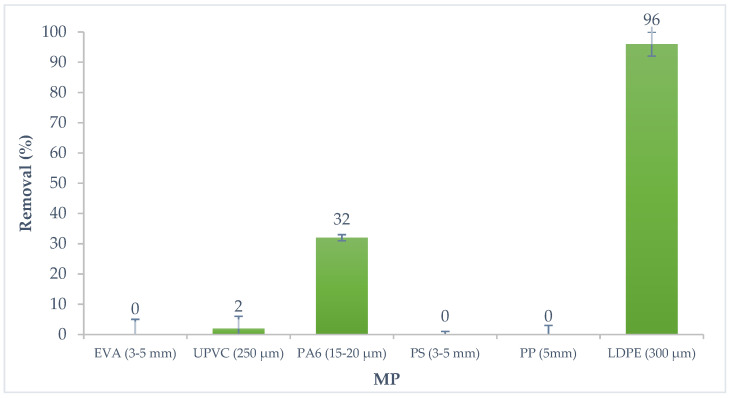
Removal of α-endosulfan onto six different MP over time.

**Figure 2:**
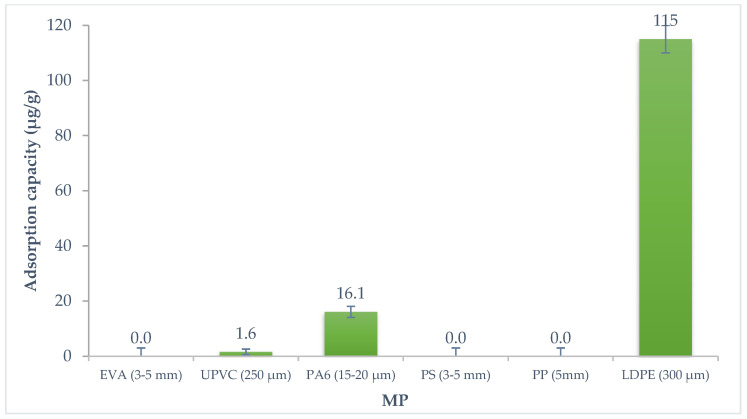
Adsorption capacity of α-endosulfan onto the six different MP.

Preliminary tests with six microplastics (concentration range 1.00–1.15 g L^−1^), with different particle sizes, were executed to evaluate the affinity of the α-endosulfan for the different MP. After a contact period of 48 h of 1 g L^−1^ of MP and 150 µg·L^−1^ of pesticide, the PA6 and LDPE adsorbed the α-endosulfan in the aqueous solution. Figure 1 presents the percentage removal of α-endosulfan for the six systems of MP studied after 48 h of contact. The adsorption capacity of each microplastic for this pesticide is shown in Figure 2.

The preliminary results showed that of the six microplastics studied in an aqueous solution with α-endosulfan, LDPE and PA6 were the ones that showed higher affinity to the pesticide, which may be related both to the chemical composition and particle size of the MP. This pesticide shows tendency to be adsorbed considering the high octanol-water partition coefficient (5.50 × 10^4^) [56]. These results demonstrate the ability of the MP to interact with other pollutants [35], such as this organochlorine pesticide. This harmful effect is aggravated by their easy spread into the environment.

The LDPE and PA6 removed a part of the pesticide, 96% (115 µg·g^−1^ adsorption capacity), and 32% (16.1 µg·g^−1^ adsorption capacity), respectively. 

PE is classified based on density and branching and they can be divided into high-density polyethylene (HDPE) and LDPE representing, respectively, 12% and 17% of the most economically important grades [57]. Fris et al. [57] and Allen et al. [58] studied the different densities of polyethylene and they conclude that LDPE presents a higher capacity to adsorb contaminants, such as PAHs and PCBs, than HDPE.

The low affinity of PA6 was also reported by Yurtsever, M. et al., [59]. In their study about the behavior of isomers α and β endosulfan in ultrapure water in the presence of PA6, the maximum adsorption capacity obtained for the PA6 in ultrapure water was 0.033 µg·g^−1^ for a 3 mm particle size. Besides this low value, this MP may have a role in the transport of this pesticide depending on the factor that influences the adsorption process, such as mixing, contact time pH, salinity, pesticide concentration, MP dosage and particle size [55]. The effect of agitation can be related to the properties of the MP, such as the floating behavior. 

The absence of removal by α-endosulfan adsorption for EVA, PS and PP was expected due to their high particle size (3 to 5 mm), shown in Table 1, since it is known that the specific surface area and the adsorption capacity increase as the particle size decreases [60]. Considering the particle size (250 μm) of UPVC, it was expected to present the capacity to adsorb α-endosulfan, however, no adsorption was observed (Figure 1 and Figure 2). Different factors can affect the adsorption capacity between the MP and contaminants, such as the glass transition temperature [60]. Polymers can be classified as glassy or rubbery according to their glass transition temperature [61]. George and Thomas [62] explain that glassy polymers have a dense structure leaving few void spaces, while rubbery polymers present a considerable free volume between molecules. MP such as PE and PP are considered rubbery plastics, and present a higher affinity for contaminants, contrary to the different types of PVC, such as UPVC or polyethylene terephthalate, which are glassy plastics [52,63]. In addition to this factor, it has been reported that the molecular structure of the PVC can also contribute to hindering the migration of contaminants into MP. Chlorine atoms are present in the PVC chains and they can introduce a polar influence, resulting in an increase in cohesive density and creating attractive forces between the individual PVC chains. This higher density provides a reduced free volume for the adsorption process when it is compared with the structure of PE [60,64].

The LDPE kinetics were explored because this MP is frequently found on the sea surface and also considering the results obtained in the preliminary experiments, which are in accordance with previous studies that demonstrate a higher affinity of polyethylene microplastics to hydrophobic compounds [3,65]. 

### 3.4. Sorption Kinetics

**Figure 3:**
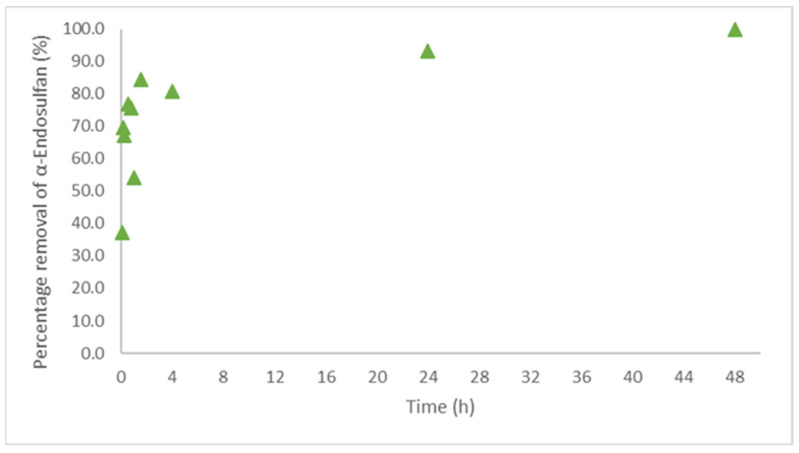
Removal of α-Endosulfan onto LDPE over time.

**Figure 4:**
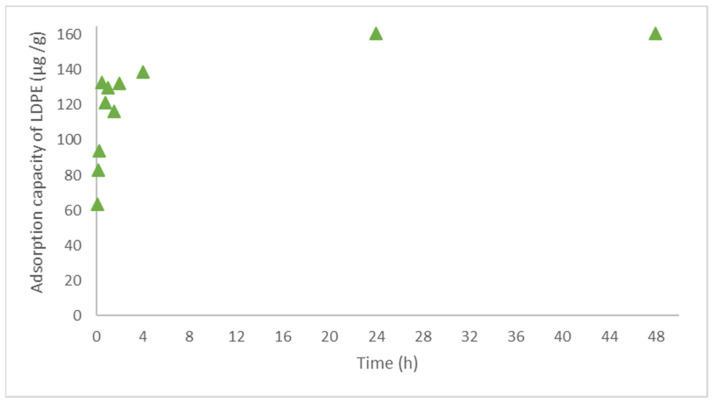
The adsorption capacity of α-endosulfan onto LDPE over time.

The kinetic studies were performed to better understand the behavior over time of the concentration of α-endosulfan in aqueous solution in the presence of LDPE. The obtained results (removal of 98% of the α-endosulfan present in the solution), shown in Figure 3 and Figure 4, are in accordance with the ones obtained in the preliminary experiments, shown in Figure 1 and Figure 2. Figure 3 illustrates the evolution during 48 h of α-endosulfan removal by LDPE (1.00 g L^−1^), complemented by Figure 4 where it is possible to observe the adsorption capacity of adsorption during that period. The sorption equilibrium time was achieved after 48 h of contact (confirmed by a new measurement after 60 h) and it is possible to observe that after 60 min, the concentration of the pesticide was reduced to half and then it slowly decreases until 48 h.

The kinetic and equilibrium studies demonstrated that LDPE could retain and transport α-endosulfan after 48 h of contact (the equilibrium time). The concentration of adsorbed pesticide in the LDPE remained stable during the next days (tested until 4 days of the experiment). Other studies with LDPE have been reporting similar equilibrium times, between 24 h and 72 h [59,66]. However, some studies mentioned that the equilibrium of PE and PP for other contaminants, such as phenanthrene, can be between 20 to 80 days [67]. It is important to point out that the environmental factors can influence the equilibrium conditions and the equilibrium may be reached more slowly than is predicted in the laboratory experiments [68].

### 3.5. Equilibrium Studies

**Figure 5:**
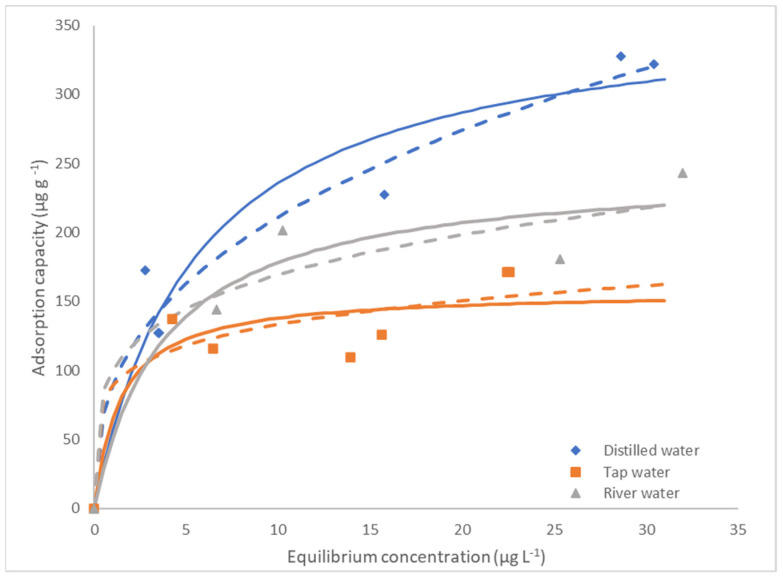
Equilibrium experimental data fitted in the Freundlich (dashed line) and Langmuir (continuous line) models for distilled, tap and filtered river waters.

Equilibrium studies were carried out for the system α-endosulfan/LDPE in different aqueous matrixes: distilled, tap, and (filtrated) river water. The experimental results and the fits of Langmuir and Freundlich equilibrium models are presented in Figure 5. The estimated models’ parameters and results of the statistical analysis are represented in Table 3. According to the results and statistical analysis, both models could represent the experimental results, although the Freundlich model presents a lower *SSE* and *χ*^2^*_Red_* and higher *R*^2^*_adj_* for distilled and tap waters and the Langmuir’s model for river water.

As in this study, there were no significant statistical differences between the Langmuir and Freundlich equilibrium models, both models could fit the experimental results (Table 3). The Freundlich model was considered the best fit in the study presented by Yurtsever, et al. [59].

As the Langmuir’s model could represent the studied systems, the estimated maximum adsorption capacities of α-endosulfan by the microplastic (q_m_) for the different systems were compared. The highest maximum adsorption capacity was obtained for distilled water, followed by the (filtered) river and tap water (i.e., 366 ± 39 µg·g^−1^; 247 ± 38 µg·g^−1^; 157 ± 22 µg·g^−1^). Comparing the maximum adsorption capacities estimated for the different types of aqueous matrices it was possible to observe that the higher adsorption capacities were observed for the simplest matrix, distilled water, followed by filtered river water and then tap water, which is related to the increase of complexity of the matrix. The increase of the matrix complexity promotes higher competition by the active sites of the adsorbent [69]. The influence of the pH also contributes to these results. The highest adsorption capacity is observed for distilled water, which presents the lowest pH (5.95), decreasing with the increase of the solution pH to around 7, in the case of filtered river water (7.22) and tap water (6.98). As in most liquid phase systems, adsorption is favored at lower pH values [69].

Comparing these results with the ones from the study of Yurtsever, et al., [59], where an adsorption capacity of 0.011 µg·g^−1^ of the isomers α and β endosulfan by LDPE (500 μm particle size) from ultrapure water was obtained, one of the biggest differences between the studies is the size of the LDPE particle, which was 300 μm in this study. The MP with a smaller particle size have a higher specific surface area available for adsorption and consequently present a higher adsorption capacity than the MP with a larger particle size [70]. For PE, which has a similar chemical composition to HDPE, Wang et al., [3] reported adsorption capacities of five different pesticides (Carbendazim, Dipterex, Diflubenzuron, Malathion, Difenoconazole) onto PE (particle size less than 5 mm), with values between 4.44 to 273.2 µg·g^−1^, which are within the range of presented results. 

## 4. Conclusions

The adsorption behavior of α-endosulfan (in different aqueous matrixes) on six types of microplastic particles, with different shapes/sizes and chemical compositions, was investigated. The obtained results showed that the adsorption process can be influenced by different factors, namely pH, particle size, matrix complexity and type of microplastic. For the LDPE/α-endosulfan system, it was possible to conclude that after 48 h of contact the equilibrium was achieved. The equilibrium study revealed that Freundlich and Langmuir’s models could represent the results. According to the Langmuir model, the highest maximum adsorption capacity was obtained for distilled water, with 366 ± 39 µg·g^−1^, followed by the (filtered) river, 247 ± 38 µg·g^−1^, and tap water with an adsorption capacity of 157 ± 22 µg·g^−1^. The highest value was observed in distilled water, followed by filtered river water and then tap water, which might be related to the lower complexity of the matrix and the lower pH. Other factors, such as the influence of microplastic aging (photo, thermal and biodegradation) should be also evaluated. Pesticides and microplastic particles coexist ubiquitously in soils, where the contact with water, and therefore the occurrence of interactions between them, such as adsorption, are possible, as demonstrated in this study, which promotes the migration of organochlorine pesticides (e.g., α-endosulfan) having microplastics (e.g., LDPE) as distribution vehicles. 

## Figures and Tables

**Table 1:** Characteristics of the studied microplastics.

MP	Particle Size	Particle Shape	Color	MP Experimental Concentration (g·L^−1^)
low-density polyethylene (LDPE)	300 μm	powder	colorless	1.00
polyethylene-co-vinyl acetate (EVA)	3–5 mm	granule	yellow	1.13
unplasticized polyvinylchloride (UPVC)	250 μm	powder	colorless	1.03
Polyamide 6 (PA6)	15–20 μm	spheroidal	colorless	1.10
polystyrene (PS)	3–5 mm	granule	colorless	1.15
polypropylene (PP)	5 mm	granule	colorless	1.01

**Table 2:** Extraction efficiency in aqueous samples (average of duplicate experiments).

Organic Solvent	C_0_ (µg·L^−1^)	Recovery Ratio (%)	RSD (%)
n-hexane	150	95	1
Ethyl acetate		86	1
Dichloromethane		68	3

**Table 3:** Equilibrium model parameters for the system α-endosulfan/LDPE, for the different aqueous matrixes, and respective statistical analyses.

Water	Freundlich					Langmuir				
	*n* (Dimensionless)	*k_F_* (µg·g^−1^ (L· μg^−1^)^1/n^)	*χ* ^2^ *_Red_*	*SSE*	*R* ^2^ *_adj_*	*q_m_* (µg·g^−1^)	*k_L_* (L· μg^−1^)	*χ* ^2^ *_Red_*	*SSE*	*R* ^2^ *_adj_*
Distilled	2.67 ± 0.38	89.0 ± 14.0	609.2	3046.2	0.955	366.4 ± 39.4	0.18 ± 0.06	1109.9	5549.7	0.919
Tap	5.72 ± 3.74	89.1 ± 27.1	498.1	2490.7	0.851	157.4 ± 22.3	0.71 ± 0.79	607.4	3037.1	0.818
Douro river	4.38 ± 2.35	100.0 ± 36.3	801.2	2403.6	0.908	246.8 ± 38.4	0.26 ± 0.19	762.1	2286.2	0.912

## Data Availability

Not applicable.

## References

[1] Wang F., Wong C.S., Chen D., Lu X., Wang F., Zeng E.Y. (2018). Interaction of toxic chemicals with microplastics: A critical review. Water Res..

[2] Pinto da Costa J., Reis V., Paço A., Costa M., Duarte A.C., Rocha-Santos T. (2019). Micro(nano)plastics—Analytical challenges towards risk evaluation. TRAC—Trend Anal. Chem..

[3] Wang T., Yu C., Chu Q., Wang F., Lan T., Wang J. (2020). Adsorption behavior and mechanism of five pesticides on microplastics from agricultural polyethylene films. Chemosphere.

[4] Rahman A., Sarkar A., Yadav O.P., Achari G., Slobodnik J. (2021). Potential human health risks due to environmental exposure to nano- and microplastics and knowledge gaps. Sci. Total Environ..

[5] Estahbanati S., Fahrenfeld N.L. (2016). Influence of wastewater treatment plant discharges on microplastic concentrations in surface water. Chemosphere.

[6] Eriksen M., Mason S., Wilson S., Box C., Zellers A., Edwards W., Farley H., Amato S. (2013). Microplastic pollution in the surface waters of the Laurentian Great Lakes. Mar. Pollut. Bull..

[7] Wu M., Yang C., Du C., Liu H. (2020). Microplastics in waters and soils: Occurrence, analytical methods and ecotoxicological effects. Ecotoxicol. Environ. Saf..

[8] Besseling E., Quik J.T.K., Sun M., Koelmans A.A. (2017). Fate of nano- and microplastic in freshwater systems: A modeling study. Environ. Pollut..

[9] Prata J.C., da Costa J.P., Lopes I., Duarte A.C., Rocha-Santos T. (2020). Environmental exposure to microplastics: An overview on possible human health effects. Sci. Total Environ..

[10] Bayo J., Olmos S., Lopez-Castellanos J. (2020). Microplastics in an urban wastewater treatment plant: The influence of physicochemical parameters and environmental factors. Chemosphere.

[11] Uddin S., Fowler S.W., Behbehani M. (2020). An assessment of microplastic inputs into the aquatic environment from wastewater streams. Mar. Pollut. Bull..

[12] Naji A., Azadkhah S., Farahani H., Uddin S., Khan F.R. (2021). Microplastics in wastewater outlets of Bandar Abbas city (Iran): A potential point source of microplastics into the Persian Gulf. Chemosphere.

[13] Reed S., Clark M., Thompson R., Hughes K.A. (2018). Microplastics in marine sediments near Rothera Research Station, Antarctica. Mar. Pollut. Bull..

[14] Lots F.A.E., Behrens P., Vijver M.G., Horton A.A., Bosker T. (2017). A large-scale investigation of microplastic contamination: Abundance and characteristics of microplastics in European beach sediment. Mar. Pollut. Bull..

[15] Wang J., Liu X., Li Y., Powell T., Wang X., Wang G., Zhang P. (2019). Microplastics as contaminants in the soil environment: A mini-review. Sci. Total Environ..

[16] Chen Y., Liu X., Leng Y., Wang J. (2020). Defense responses in earthworms (Eisenia fetida) exposed to low-density polyethylene microplastics in soils. Ecotoxicol. Environ. Saf..

[17] Cole M., Lindeque P., Halsband C., Galloway T.S. (2011). Microplastics as contaminants in the marine environment: A review. Mar. Pollut. Bull..

[18] Du J., Xu S., Zhou Q., Li H., Fu L., Tang J., Wang Y., Peng X., Xu Y., Du X. (2020). A review of microplastics in the aquatic environmental: Distribution, transport, ecotoxicology, and toxicological mechanisms. Environ. Sci. Pollut. Res. Int..

[19] Chen Q., Allgeier A., Yin D., Hollert H. (2019). Leaching of endocrine disrupting chemicals from marine microplastics and mesoplastics under common life stress conditions. Environ. Int..

[20] Kwon J.H., Kim J.W., Pham T.D., Tarafdar A., Hong S., Chun S.H., Lee S.H., Kang D.Y., Kim J.Y., Kim S.B. (2020). Microplastics in Food: A Review on Analytical Methods and Challenges. Int. J. Environ. Res. Public Health.

[21] Uddin S., Fowler S.W., Habibi N., Sajid S., Dupont S., Behbehani M. (2022). A Preliminary Assessment of Size-Fractionated Microplastics in Indoor Aerosol-Kuwait’s Baseline. Toxics.

[22] Habibi N., Uddin S., Fowler S.W., Behbehani M. (2022). Microplastics in the atmosphere: A review. JEEA.

[23] Selonen S., Dolar A., Jemec Kokalj A., Sackey L.N.A., Skalar T., Cruz Fernandes V., Rede D., Delerue-Matos C., Hurley R., Nizzetto L. (2021). Exploring the impacts of microplastics and associated chemicals in the terrestrial environment—Exposure of soil invertebrates to tire particles. Environ. Res..

[24] Abbasi S., Soltani N., Keshavarzi B., Moore F., Turner A., Hassanaghaei M. (2018). Microplastics in different tissues of fish and prawn from the Musa Estuary, Persian Gulf. Chemosphere.

[25] Barboza L.G.A., Lopes C., Oliveira P., Bessa F., Otero V., Henriques B., Raimundo J., Caetano M., Vale C., Guilhermino L. (2020). Microplastics in wild fish from North East Atlantic Ocean and its potential for causing neurotoxic effects, lipid oxidative damage, and human health risks associated with ingestion exposure. Sci. Total Environ..

[26] Browne M.A., Dissanayake A., Galloway T.S., Lowe D.M., Thompson R.C. (2008). Ingested microscopic plastic translocates to the circulatory system of the mussel, Mytilus edulis (L). Environ. Sci. Technol..

[27] Mohsen M., Wang Q., Zhang L., Sun L., Lin C., Yang H. (2019). Microplastic ingestion by the farmed sea cucumber Apostichopus japonicus in China. Environ. Pollut..

[28] Vroom R.J.E., Koelmans A.A., Besseling E., Halsband C. (2017). Aging of microplastics promotes their ingestion by marine zooplankton. Environ. Pollut..

[29] Zhang S., Wang J., Liu X., Qu F., Wang X., Wang X., Li Y., Sun Y. (2019). Microplastics in the environment: A review of analytical methods, distribution, and biological effects. TRAC—Trend Anal. Chem..

[30] Zakeri M., Naji A., Akbarzadeh A., Uddin S. (2020). Microplastic ingestion in important commercial fish in the southern Caspian Sea. Mar. Pollut. Bull..

[31] Devriese L.I., van der Meulen M.D., Maes T., Bekaert K., Paul-Pont I., Frere L., Robbens J., Vethaak A.D. (2015). Microplastic contamination in brown shrimp (Crangon crangon, Linnaeus 1758) from coastal waters of the Southern North Sea and Channel area. Mar. Pollut. Bull..

[32] Li J., Lusher A.L., Rotchell J.M., Deudero S., Turra A., Brate I.L.N., Sun C., Shahadat Hossain M., Li Q., Kolandhasamy P. (2019). Using mussel as a global bioindicator of coastal microplastic pollution. Environ. Pollut..

[33] Lusher A.L., Welden N.A., Sobral P., Cole M. (2017). Sampling, isolating and identifying microplastics ingested by fish and invertebrates. Anal. Methods.

[34] Rochman C.M., Hoh E., Kurobe T., Teh S.J. (2013). Ingested plastic transfers hazardous chemicals to fish and induces hepatic stress. Sci. Rep..

[35] Sussarellu R., Suquet M., Thomas Y., Lambert C., Fabioux C., Pernet M.E., Le Goic N., Quillien V., Mingant C., Epelboin Y. (2016). Oyster reproduction is affected by exposure to polystyrene microplastics. Proc. Natl. Acad. Sci. USA.

[36] Prata J.C., da Costa J.P., Lopes I., Duarte A.C., Rocha-Santos T. (2020). Environmental status of (micro)plastics contamination in Portugal. Ecotoxicol. Environ. Saf..

[37] Zhou B., Wang J., Zhang H., Shi H., Fei Y., Huang S., Tong Y., Wen D., Luo Y., Barcelo D. (2020). Microplastics in agricultural soils on the coastal plain of Hangzhou Bay, east China: Multiple sources other than plastic mulching film. J. Hazard. Mater..

[38] Li W., Wufuer R., Duo J., Wang S., Luo Y., Zhang D., Pan X. (2020). Microplastics in agricultural soils: Extraction and characterization after different periods of polythene film mulching in an arid region. Sci. Total Environ..

[39] Hurley R.R., Nizzetto L. (2018). Fate and occurrence of micro(nano)plastics in soils: Knowledge gaps and possible risks. Curr. Opin. Environ. Sci. Health.

[40] Bussian B.M., Pandelova M., Lehnik-Habrink P., Aichner B., Henkelmann B., Schramm K.W. (2015). Persistent endosulfan sulfate is found with highest abundance among endosulfan I, II, and sulfate in German forest soils. Environ. Pollut..

[41] Allinson G., Allinson M., Bui A., Zhang P., Croatto G., Wightwick A., Rose G., Walters R. (2016). Pesticide and trace metals in surface waters and sediments of rivers entering the Corner Inlet Marine National Park, Victoria, Australia. Environ. Sci. Pollut. Res. Int..

[42] Roberts D.M., Karunarathna A., Buckley N.A., Manuweera G., Sheriff M.H., Eddleston M. (2003). Influence of pesticide regulation on acute poisoning deaths in Sri Lanka. Bull. World Health Organ..

[43] Mathanakeerthi S., Sadheesh S., Nandha kumar M., Gowtham S., Manoj Kumar V. (2021). Adsorption of endosulfan from aqueous solution using graphene clay matrix (GCM). Mater. Today Proc..

[44] Weber J., Halsall C.J., Muir D., Teixeira C., Small J., Solomon K., Hermanson M., Hung H., Bidleman T. (2010). Endosulfan, a global pesticide: A review of its fate in the environment and occurrence in the Arctic. Sci. Total Environ..

[45] Mudhoo A., Bhatnagar A., Rantalankila M., Srivastava V., Sillanpää M. (2019). Endosulfan removal through bioremediation, photocatalytic degradation, adsorption and membrane separation processes: A review. Chem. Eng. Jorn..

[46] Vera J., Fernandes V.C., Correia-Sá L., Mansilha C., Delerue-Matos C., Domingues V.F. (2021). Occurrence of Selected Known or Suspected Endocrine-Disrupting Pesticides in Portuguese Surface Waters Using SPME-GC-IT/MS. Separations.

[47] Paiga P., Sousa S., Vera J., Bitencourt L., Vieira J., Jorge S., Silva J.G., Correia M., Domingues V.F., Delerue-Matos C. (2021). Multi-residue analysis of fifty pesticides in river waters and in wastewaters. Environ. Sci. Pollut. Res. Int..

[48] Rodrigues J.P., Duarte A.C., Santos-Echeandía J., Rocha-Santos T. (2019). Significance of interactions between microplastics and POPs in the marine environment: A critical overview. TRAC—Trend Anal. Chem..

[49] Lee H., Byun D.-E., Kim J.M., Kwon J.-H. (2018). Desorption of Hydrophobic Organic Chemicals from Fragment-Type Microplastics. Ocean Sci..

[50] Chen S., Tan Z., Qi Y., Ouyang C. (2019). Sorption of tri-n-butyl phosphate and tris(2-chloroethyl) phosphate on polyethylene and polyvinyl chloride microplastics in seawater. Mar. Pollut. Bull..

[51] Xu P., Ge W., Chai C., Zhang Y., Jiang T., Xia B. (2019). Sorption of polybrominated diphenyl ethers by microplastics. Mar. Pollut. Bull..

[52] Guo X., Wang X., Zhou X., Kong X., Tao S., Xing B. (2012). Sorption of four hydrophobic organic compounds by three chemically distinct polymers: Role of chemical and physical composition. Environ. Sci. Technol..

[53] Wang J., Peng J., Tan Z., Gao Y., Zhan Z., Chen Q., Cai L. (2017). Microplastics in the surface sediments from the Beijiang River littoral zone: Composition, abundance, surface textures and interaction with heavy metals. Chemosphere.

[54] Weber J. (1972). Physicochemical Process for Water Quality Control.

[55] Tang S., Lin L., Wang X., Sun X., Yu A. (2021). Adsorption of fulvic acid onto polyamide 6 microplastics: Influencing factors, kinetics modeling, site energy distribution and interaction mechanisms. Chemosphere.

[56] Endosulfan. Monograph Annex B 2-: Physical and Chemical Properties Monograph July 1999; III Chapter 2. http://chm.pops.int/portals/0/docs/from_old_website/documents/meetings/poprc/submissions/Endosulfan_2008/Endosulfan_EU_ADDENDUM_B2_%20July2001-san.pdf.

[57] Fries E., Zarfl C. (2012). Sorption of polycyclic aromatic hydrocarbons (PAHs) to low and high density polyethylene (PE). Environ. Sci. Pollut. Res. Int..

[58] Allen T., Farley S., Draper J., Clement C., Polidoro B. (2018). Variations in Sorption of Organochlorine Pesticides and PCBs across Six Different Plastic Polymers. J. Environ. Toxicol. Stud..

[59] Yurtsever M., Oz N., Aksu A., Balkis N., Altug G., Taskin O.S. (2020). Hydrophobic Pesticide Endosulfan (α + β) and Endrin Sorption on Different Types of Microplastics. J. Chem. Soc. Pak..

[60] Wang F., Zhang M., Sha W., Wang Y., Hao H., Dou Y., Li Y. (2020). Sorption Behavior and Mechanisms of Organic Contaminants to Nano and Microplastics. Molecules.

[61] Teuten E.L., Saquing J.M., Knappe D.R., Barlaz M.A., Jonsson S., Bjorn A., Rowland S.J., Thompson R.C., Galloway T.S., Yamashita R. (2009). Transport and release of chemicals from plastics to the environment and to wildlife. Philos. Trans. R. Soc. Lond. B Biol. Sci..

[62] George S.C., Thomas S. (2001). Transport phenomena through polymeric systems. Prog. Polym. Sci..

[63] Wu B., Taylor C.M., Knappe D.R., Nanny M.A., Barlaz M.A. (2001). Factors controlling alkylbenzene sorption to municipal solid waste. Environ. Sci. Technol..

[64] Pascall M.A., Zabik M.E., Zabik M.J., Hernandez R.J. (2005). Uptake of polychlorinated biphenyls (PCBs) from an aqueous medium by polyethylene, polyvinyl chloride, and polystyrene.e films. J. Agric. Food Chem..

[65] Wang F., Gao J., Zhai W., Liu D., Zhou Z., Wang P. (2020). The influence of polyethylene microplastics on pesticide residue and degradation in the aquatic environment. J. Hazard. Mater..

[66] Puckowski A., Cwiek W., Mioduszewska K., Stepnowski P., Bialk-Bielinska A. (2021). Sorption of pharmaceuticals on the surface of microplastics. Chemosphere..

[67] Karapanagioti H.K., Klontza I. (2008). Testing phenanthrene distribution properties of virgin plastic pellets and plastic eroded pellets found on Lesvos island beaches (Greece). Mar. Environ. Res..

[68] Mato Y., Isobe T., Takada H., Kanehiro H., Ohtake C., Kaminuma T. (2001). Plastic Resin Pellets as a transport Medium for Toxic Chemicals in the Marine Environment. Environ. Sci. Technol..

[69] Edeline F. (1998). L’épuration Physico-Chimique des Eaux: Théorie et Technologie.

[70] Gao X., Hassan I., Peng Y., Huo S., Ling L. (2021). Behaviors and influencing factors of the heavy metals adsorption onto microplastics: A review. J. Clean. Prod..

